# Adherence to secondary prophylaxis for rheumatic heart disease is underestimated by register data

**DOI:** 10.1371/journal.pone.0178264

**Published:** 2017-05-31

**Authors:** Jessica Langloh de Dassel, Marea Therese Fittock, Sagen Cheyenne Wilks, Jane Elizabeth Poole, Jonathan Rhys Carapetis, Anna P. Ralph

**Affiliations:** 1 Institute of Advanced Studies, Charles Darwin University, Darwin, Northern Territory, Australia; 2 Menzies School of Health Research, Darwin, Northern Territory, Australia; 3 Northern Territory Rheumatic Heart Disease Control Program, Northern Territory Department of Health, Darwin, Northern Territory, Australia; 4 Telethon Kids Institute, University of Western Australia, Perth, Western Australia, Australia; 5 Perth Children’s Hospital, Perth, Western Australia, Australia; 6 Royal Darwin Hospital, Darwin, Northern Territory, Australia; University of Bologna, ITALY

## Abstract

**Objective:**

In high-burden Australian states and territories, registers of patients with acute rheumatic fever and rheumatic heart disease are maintained for patient management, monitoring of system performance and research. Data validation was undertaken for the Australian Northern Territory Rheumatic Heart Disease Register to determine quality and impact of data cleaning on reporting against key performance indicators: overall adherence, and proportion of patients receiving ≥80% of scheduled penicillin doses for secondary prophylaxis.

**Methods:**

Register data were compared with data from health centres. Inconsistencies were identified and corrected; adherence was calculated before and after cleaning.

**Results:**

2780 penicillin doses were validated; 426 inconsistencies were identified, including 102 incorrect dose dates. After cleaning, mean adherence increased (63.5% to 67.3%, p<0.001) and proportion of patients receiving ≥80% of doses increased (34.2% to 42.1%, p = 0.06).

**Conclusions:**

The Northern Territory Rheumatic Heart Disease Register underestimates adherence, although the key performance indicator of ≥80% adherence was not significantly affected. Program performance is better than hitherto appreciated. However some errors could affect patient management, as well as accuracy of longitudinal or inter-jurisdictional comparisons. Adequate resources are needed for maintenance of data quality in acute rheumatic fever/rheumatic heart disease registers to ensure provision of evidence-based care and accurate assessment of program impact.

## Introduction

Acute rheumatic fever and rheumatic heart disease have been eliminated as public health problems from high-income settings, however they continue to cause significant morbidity and premature mortality in low income settings, including Australia’s Indigenous population.[1] A key evidence-based public health focus for people living with ARF or RHD (PLWRHD) is the provision of secondary prophylaxis: injections of benzathine penicillin G (BPG) every 28 days to prevent ARF recurrences and development or progression of RHD.[2] A key performance indicator (KPI) is the proportion of individuals receiving ≥80% of scheduled injections. The World Heart Federation recommends the establishment of register-based control programs to facilitate the coordination of care and follow up for PLWRHD[3]; these programs have enhanced the delivery of secondary prophylaxis in several settings[4].

The Northern Territory (NT) RHD Control Program (Control Program) maintains the jurisdictional register (Register)–a secured online database containing information on the provision of care to PLWRHD which can be accessed only by authorised health professionals and researchers. Register data facilitate the provision of care to PLWRHD; enable monitoring of ARF and RHD control at clinic and jurisdiction levels, and are fed into a centralised data collection system which reports against KPIs to the Commonwealth Government.[5] High data quality is crucial for the appropriate management of individuals living with ARF and RHD, and for continued improvement of ARF and RHD control activities. The Register enables the compilation and storage of relevant data including information from primary health centres, tertiary hospitals and specialists; however, the Register is not the primary patient record system for PLWRHD living in the Northern Territory.

Health services document the provision of BPG to PLWRHD in their patient information reporting system; this information is communicated to the Control Program by faxed handwritten ‘mastercharts’, or through electronic reports which may be generated and dispatched by clinic staff or automatically produced and submitted. Data are entered manually onto the Register by Control Program staff. The Register software provides no feedback on allowable values when data are entered and the Control Program team have limited resources to conduct systematic data reviews. Only one prior study has investigated the integrity of the Register data; it was conducted in one community only, and occurred prior to the introduction of an electronic patient information record system at primary health centres. The study found that that 19% of the PLWRHD in that community had not been included in the Register. Forty two percent of the PLWRHD at this community were receiving at least 80% of the scheduled BPG injections.[6] This study did not validate BPG dose data.

The Register data are used extensively by researchers to inform research priorities and answer epidemiological questions.[6–11] Persistent suboptimal secondary prophylaxis adherence rates in the NT demonstrated by the Register prompted the development of a large community trial which aims to improve secondary prophylaxis for ARF and RHD.[12] Register data will be used to demonstrate the trial’s impact. To ensure the findings are accurate, systematic cleaning of the Register data was undertaken.

We sought to develop and implement a data cleaning process to cross-check recording of BPG doses in the Register against primary data recorded by clinics. This article describes the cleaning procedure, reports the impact of cleaning on indicators of adherence and discusses the clinical, research and programmatic implications.

## Methods

### Data extraction

Data for 1 December 2012 through 31 August 2014 were extracted from the Register by the Data Officer and provided in password-protected files. Fields extracted from the Register included: unique patient identifier (hospital record number, (HRN)), date of birth, sex, secondary prophylaxis start and cease dates, BPG administration dates, clinic where the BPG was administered. Identifying fields were used for data matching purposes only.

### Ethical approval

The study was approved by the Human Research Ethics Committee of the Northern Territory Department of Health and Menzies School of Health Research (2012–1756) and the Central Australian Human Research Ethics Committee (13–126). The ethics approvals waived the requirement for individual consent. The authors had access to identifying information, specifically for the purpose of data validation.

### Data review process

Processes for data review were decided by consensus of study investigators including a statistician. Initially, ten randomly selected patients from each of ten communities were selected for cleaning. The dose date and the name of the clinic where the dose was administered (‘dose clinic’) recorded in the Register were compared with primary data sources, i.e. the health centre databases. Inconsistencies were documented in a spreadsheet and discussed with Control Program staff; the Register and trial dataset were corrected as needed. Overall error rates were calculated for each site using the formula:

$$\mathrm{error}\;\mathrm{rate}\;=\frac{\mathrm{total}\;\mathrm{number}\;\mathrm{of}\;\mathrm{errors}}{\mathrm{number}\;\mathrm{of}\;\mathrm{BPG}\;\mathrm{doses}\;\mathrm{validated}}\times 100$$

If a dose was missing from the Register, two errors were recorded: one for the missing dose date and one for the missing dose clinic location. If the error rate was >10% all patients at that site were then included for validation. After further consideration, the team recognised that two data fields (the dose date and the clinic location) were being validated for each BPG dose, and doses missing from the Register needed to be included in the denominator. The error rate calculation was revised to:

$$\mathrm{error}\;\mathrm{rate}\;=\frac{\mathrm{total}\;\mathrm{number}\;\mathrm{of}\;\mathrm{errors}}{(\mathrm{number}\;\mathrm{of}\;\mathrm{BPG}\;\mathrm{doses}\;\mathrm{validated}\;+\mathrm{number}\;\mathrm{of}\;\mathrm{missing}\;\mathrm{BPG}\;\mathrm{doses}\;\mathrm{identified})\times 2}\times 100$$

The revised formula was used to calculate the error rates reported in Table 1. Date-specific error rates were also calculated (errors in location do not affect adherence indicators). Data relating to BPG administered at hospitals, interstate or at community-controlled sites not participating in the trial, could not be validated because the study team did not have access to those databases.

**Table 1:** Adherence before and after validation.

					Adherence rate (%)		Proportion of patients receiving 80% or more of scheduled injections (%)	
Site	Patients in final validation sample	Doses missing from Register (n)	Total error rate (%)*	Date error rate (%)	Raw data	Corrected data	Raw data	Corrected data
**Site 1**	10	2	6	3.1	42	43	0	0
**Site 2**	21	39	17.8	9.1	70	82	33	62
**Site 3**	10	1	5.9	4.7	73	73	60	60
**Site 4**	41	39	10.7	6.4	52	56	12	22
**Site 5**	10	0	2.9	2.6	70	70	50	50
**Site 6**	13	11	8.8	4.4	65	67	23	23
**Site 7**	10	1	3.5	3.1	74	74	50	50
**Site 8**	34	36	9.1	6.6	77	81	59	71
**Site 9**	78	63	12.7	6.4	50	53	21	24
**Site 10**	10	4	4.4	2.2	72	73	50	60
**Total**	**237**	**196**						
**Mean**			**8**	**6**	**63.5**	**67.3**	**34.2**	**42.1**

* revised error rate formula used.

Several rules were developed and applied to inconsistent data points:

1. If a BPG dose was documented in the health centre database but was missing from the RHD Register, this BPG dose was added to the Register and the study dataset.
2. If the date of a BPG dose in the Register did not match the health centre database, the date in the health centre database was used and the Register was corrected.
3. A BPG dose documented in the Register but not documented in the health centre databases was kept in the Register and the study dataset.
4. If the BPG clinic location did not match, the location documented in the health centre database was used.

### Calculations and analyses

Site specific adherence rates were calculated before and after cleaning using the formula:

$$\mathrm{adherence}\;\mathrm{rate}=\frac{\mathrm{total}\;\mathrm{number}\;\mathrm{of}\;\mathrm{doses}\;\mathrm{delivered}}{(\mathrm{total}\;\mathrm{number}\;\mathrm{of}\;\mathrm{days}⁄28)\times \mathrm{number}\;\mathrm{of}\;\mathrm{individuals}\;\mathrm{in}\;\mathrm{the}\;\mathrm{validated}\;\mathrm{dataset}}$$

The denominator was adjusted for patients whose secondary prophylaxis prescription was discontinued during the timeframe.

A decision rule was developed whereby doses were retained in the Register even in the absence of supporting primary documentation, since primary documentation could have occurred in a database inaccessible to study staff (e.g. a hospital database).

Statistical analyses were undertaken using Stata 14.0 (College Station, Texas 77845 USA). Change in average adherence rate was estimated using a linear mixed effect model that accounted for potential clustering of observations at the clinic and patient level. The change in the proportion of patients receiving at least 80% of the scheduled injections was estimated using a mixed effects logistic regression model that accounted for the potential clustering of observations at the clinic level.

## Results

The data extracted from the Register included 7477 penicillin doses, which were administered to 429 patients (Table 1). Validation of the complete patient list was required for four sites (the original error rate formula was used to determine whether the complete patient list required validation; the error rates reported in Table 1 were calculated using the revised formula). Data for 237 patients (including 2780 penicillin doses) were validated; 353 doses (11.3%) in the validation subset could not be validated due to limited access to health centre and hospital databases.

### Inconsistencies

Sixty six doses were recorded in the Register but not in the health centre databases; 196 doses were recorded in health centre databases but not in the Register; these were added to the Register. All doses were included in the study dataset. One hundred and two dose dates in the Register did not match the health centre data. The mean overall error rate was 8% (range: 3%–18%); the mean date error rate was 6% (range: 2%–9%).

### Impact on adherence rates

After data cleaning the adherence rate increased at nine sites; it was unchanged at one site (Table 1). Mean adherence calculated using the raw data was 63.5%; using the cleaned data mean adherence was significantly higher (67.3%, p<0.001).

The proportion of patients receiving ≥80% of scheduled injections increased at five sites after validation; it remained unchanged at five sites. The change in mean proportion of patients receiving ≥80% was not statistically significant (34.2% vs 42.1%, OR = 1.49 (95% CI: 0.98–2.25), p = 0.06).

The dataset is provided in S1 File, and the Stata code is contained in S2 File.

## Discussion

We have found that in a large disease register requiring manual data entry, data accuracy was high overall, but data errors were identified which resulted in under-estimation of adherence. The proportion of patients receiving ≥80% of scheduled penicillin doses, an important KPI, was not significantly affected by data cleaning (Table 1), but is likely to have been had the sample been larger (our sample accounts for approximately 18% of the total number of people on secondary prophylaxis between 1 December 2012 and 31 August 2014). However data cleaning resulted in a statistically significant increase in overall adherence, relevant for both programmatic feedback and research purposes. Data errors may have been caused by the Register software, illegible handwriting on a ‘masterchart’, inappropriate documentation of the BPG administration in the health centres’ databases such that it was not reported to the Register, or human error during data entry.

Given the context of remote health and the limited resources available to the NT Control Program, the overall high level of data accuracy is heartening—only five fulltime equivalent staff are employed to support the clinical care of over 2000 individuals living with ARF or RHD, provide education and advice to health professionals working in more than 100 remote health centres and three tertiary centres, and manage the Register. Nevertheless, the potential impacts of the level of error we have identified are important and may negatively affect individual patient care, monitoring of RHD Care performance overall and morale of health centre staff, due to underestimation of performance. The dose administration date was incorrect in 3.7% of cases. While most health centres use their own patient data to determine when the next BPG injection is due, in some situations the Register data may be used (for example when a person with ARF or RHD is admitted to hospital or prison, or travels to another community, and their primary health care information can not be easily accessed). In these instances, incorrect and missing dose dates in the Register could result in a patient receiving their next injection too late or unnecessarily early. Delayed injections would provide inadequate protection from streptococcal infection and risk of ARF recurrence with further damage to cardiac valves.[9] Over-treatment could cause frustration for patients, who in this context often lack agency to decline treatment: a mutually trusting and respectful relationship between healthcare providers and patients is needed for patients to have confidence to question the timing of doses being recommended to them, however the challenges faced by health providers and patients in this setting mean this kind of relationship is not always achieved. BPG injections can have occasional adverse side effects[13] and excess treatment could lead to the patient becoming disengaged and less adherent. Accuracy of dose dates within the centralised register is even more important for communities where calendar literacy may be low and mechanisms to record dose dates may not be available for individuals. Traditionally, moon phases have been used as a guide for patients to recall when their needle is due; calendars and reminder cards are also used, and a smartphone application is also now available.[14]

Disease registers are widely acknowledged as valuable mechanisms to improve patient care, benchmark outcomes, compare performance longitudinally and across sites, and provide data for research and national reporting.[15] In Australia, jurisdictional data from RHD Control Programs are provided to the centralised Data Collection System and also to the Australian Institute of Health and Welfare for periodic reporting.[16] Internationally, the absence of systematically collected recent data on RHD treatment and outcomes has been identified as a key gap in our understanding of ARF and RHD management, and as a result the REMEDY registry (*R*h*e*u*m*atic H*e*art *D*isease Registr*y*) has been initiated.[17] REMEDY currently has data on 3343 people with RHD from Africa, the Middle East and India and includes information on adherence to secondary prophylaxis.[18]

Achievements of the register–based NT RHD Control Program include an increase in delivery of secondary prophylaxis from only 21% of patients receiving ≥80% of annual scheduled doses in 2008 to >45% now (personal communication, Marea Fittock, NT RHD Control Program) and development of the ‘days at risk’ concept. ‘Days at risk’ refers to days when a patient is not protected from a GAS infection, which happens when a dose of penicillin is delayed. For pragmatic purposes, days at risk are considered to commence on the 29^th^ day after a dose of BPG is administered, and continue until the next dose is provided. The NT RHD Control Program team have explained days at risk to health centre staff throughout the Northern Territory. The rollout of this concept has been accompanied by a change in the timing of the patient recall for BPG: previously the recall process commenced on day 28, when the dose was due to be delivered. In many instances it could take several days for a patient to be located and for the patient to present to the health centre, resulting in a delay in the provision of the penicillin. The recall process in most NT health centres now starts on the 21^st^ day after the previous dose, providing a seven day window for the penicillin to be administered on time.[9]

The current study indicates that there is room to improve the accuracy of the Register data. A planned software upgrade and the automation of the entry of BPG administration data should enhance the quality of BPG data. To maximise data quality all health professionals could be supported to consistently document BPG administration using the recommended database fields for that clinic and regular comparisons of clinic data with the Register would assist with the identification of any persisting documentation issues. With respect to the Register software, electronic reports could be tested to ensure the relevant data are extracted and transmitted to the Register; expanded functionality to enable the documentation of changes to an individual’s secondary prophylaxis regimen over time would also increase the accuracy of adherence calculations.

Limitations of this study include that some primary source data were inaccessible to the research team; while the proportion of injection doses unable to be validated was low, it is possible that there are additional doses which have not been recorded on the Register, and that actual adherence is even higher than reported here. Also, we validated data on secondary prophylaxis only; the Register also contains extensive information on each patient’s RHD severity and aspects of clinical care which we did not check. However, the primary function of ARF/RHD registers in many settings is to coordinate delivery of secondary prophylaxis,[4] hence the BPG administration date is the most important field to validate. Finally, the study was restricted to ten communities which had consented to their databases being accessed by the research team; however the Control Program is validating Register data from all health centres in the NT which will further increase the accuracy of the data and the indicators calculated using the database.

## Conclusion

This study found that using the Register data to calculate adherence rates could lead to an underestimation of the provision of secondary prophylaxis in the Northern Territory. The data corrections we made have contributed to the improvement of data quality. To our knowledge this is the first time the process and outcomes of the validation of RHD data have been published. Accurate data are crucial for the provision of evidence-based care for people living with ARF and RHD in the NT. In addition, assessment of the impact of the NT RHD Control Program needs to be made using high quality data. The findings of this validation exercise suggest ongoing data review and validation could be valuable. These tasks will be facilitated with the introduction of automated data checks which will be incorporated in the upcoming upgrade to the Register software. Improved accuracy of secondary prophylaxis data is likely to enhance individual patient care and the monitoring of the performance of the health system. In addition, the documentation and subsequent reporting of the actual level of care provided by health services should improve staff morale.

### Implications for public health

Settings with high ARF/RHD burdens require well-staffed, high-quality disease registers as recommended by the World Health Organization. A register-based control program is the best mechanism to address the challenge of coordinating delivery of regular BPG injections and other aspects of patient care for this serious but relatively rare condition. Adequate resources are needed for maintenance of high-quality ARF/RHD registers to ensure provision of evidence-based care and accurate assessment of program impact.

## Supporting information

S1 File Rheumatic heart disease prophylaxis adherence validation dataset. (XLSX)

S2 File Rheumatic heart disease prophylaxis adherence Stata code. (DOCX)
