# Supplementary material for: Adherence to secondary prophylaxis for rheumatic heart disease is underestimated by register data
Source: PLoS One. 2017 May 31;12(5):e0178264. doi: 10.1371/journal.pone.0178264 (PMC5451029; doi:10.1371/journal.pone.0178264)
Supplement: S2 File — (DOCX) [file pone.0178264.s002.docx]

**S2 File**

**Rheumatic heart disease prophylaxis adherence Stata code**

egen clust=group(clinic patientid)

xtset newid time

melogit adhcat i.time || clinic: , or

margins i.time
